# Anti‐HBc‐nonreactive occult hepatitis B infections with HBV genotypes B and C in vaccinated immunocompetent adults

**DOI:** 10.1111/jvh.13733

**Published:** 2022-08-24

**Authors:** Xuelian Deng, Xiaohan Guo, Hongfang Gu, Dong Wang, Syria Laperche, Jean‐Pierre Allain, Liang Zang, Daniel Candotti

**Affiliations:** ^1^ Dalian Blood Center Dalian China; ^2^ Dalian Public Health Clinical Center Dalian China; ^3^ Department of Blood Transmitted Agents National Institute of Blood Transfusion Paris France; ^4^ University of Cambridge Cambridge UK; ^5^ Department of Virology Henri Mondor Hospital, AP‐HP Créteil France; ^6^ University of Paris‐Est, INSERM U955, IMRB Créteil France

**Keywords:** anti‐HBs, blood donors, HBV variants, hepatitis B vaccine, hepatitis B virus, occult infection, viral DNA

## Abstract

Absence of anti‐HBc reactivity with detectable anti‐HBs was observed in blood donors with occult hepatitis B virus (HBV) infection (OBI). The prevalence and mechanisms underlying this uncommon condition were investigated over time in Chinese blood donors with OBI. Isolated anti‐HBs OBI status was identified from 466,911 donors from Dalian, China, and monitored in follow‐up (range: 2.6–84.3 months). HBV vaccination status was documented, and infecting viral strains were characterized. Of 451 confirmed OBIs (1:1035), 43 (9.5%; 1:10,858) had isolated anti‐HBs as only serological marker. Isolated anti‐HBs OBIs differed from anti‐HBc‐reactive OBIs by significantly younger age (median 24 years), higher HBV DNA (median: 20 IU/ml) and anti‐HBs (median 60.5 IU/L) levels, paucity of mutations in HBV Core and S proteins, and high vaccination rate (72%). Vaccinated isolated anti‐HBs OBIs (*n* = 31) differed from unvaccinated (*n* = 11) by significantly younger age (22 vs 38 years), higher anti‐HBs level at index (48% vs 9% with anti‐HBs >100 IU/L) and higher frequency of anti‐HBs immune response (44% vs 20%). Of 15 vaccinated and 5 unvaccinated OBIs follow‐up, 65% (8 vaccinated and 5 unvaccinated) became HBV DNA negative suggesting aborted recent infection, while 35% (7 vaccinated) had low persistent viraemia 2 to 65 months post index. In conclusion, isolated anti‐HBs OBI in Chinese blood donors appears associated with young, vaccinated, adults exposed to HBV who predominantly develop low level aborted infection revealed by transient HBV DNA and immune anti‐HBs response. However, a subset of individuals still experienced low but persistent viral replication whose clinical outcome remains uncertain.

## 1 INTRODUCTION

In recent years, occult hepatitis B infection (OBI) has been identified as a particular feature of the hepatitis B virus (HBV) infection natural history. OBI is characterized by the presence of generally low levels of replication‐competent HBV DNA in the liver and/or in the blood without detectable HBsAg.^1^ OBI is mostly associated with anti‐HBc that is usually detectable 6–12 weeks after infection and remains detectable lifelong in immunocompetent individuals when anti‐HBs level declines to undetectable levels and HBV DNA is often intermittently detectable. Unusual combinations of anti‐HBc and anti‐HBs profiles were observed in OBI carriers that may lead to uncertainty in the interpretation of results or suspicion of testing errors. A multiregional study reported anti‐HBc‐negative OBI in 0.6% of asymptomatic blood donors, 83% of whom carried anti‐HBs as the only serological marker.^2^ Significantly lower frequencies were observed in OBI blood donors from Europe (2.4%), the Mediterranean region (4.3%) and South Africa (6.2%). This difference in isolated anti‐HBs prevalence remains unclear but may be related to regional differences in HBV endemicity, prophylaxis programs, natural history of HBV infection and circulating HBV genotypes.

Rare cases of anti‐HBc‐negative overt chronic HBV infection have been documented, mainly in immunocompromised patients experiencing HBV reactivation or subjects co‐infected with HIV, patients infected with HBV variants carrying mutations that might impair HBcAg expression or detection, and infants born to HBsAg‐positive mothers.^3^, ^4^, ^5^, ^6^ In China and South East Asia, isolated anti‐HBs were reported in 6.3%–20.3% of immunocompetent blood donors with OBI.^2^, ^7^, ^8^, ^9^ Isolated anti‐HBs OBI has been also reported in immunized neonates born to HBsAg‐positive mothers and children with HBsAg‐positive parents.^10^, ^11^, ^12^, ^13^, ^14^ When available, follow‐up of vaccinated children in these studies was relatively limited in time and often showed low and transient HBV DNA levels.^11^, ^13^ In contrast, a Taiwanese cross‐sectional study reported HBV viraemia in fully immunized individuals aged >18 years who were negative for anti‐HBc.^10^ Further, vaccinated blood donors sexually exposed to partners with chronic HBV developed anti‐HBc‐negative OBI reflecting an aborted form of infection.^15^

The host and/or viral mechanisms leading to isolated anti‐HBs OBI and the evolution of this condition and its potential clinical outcome remain largely unknown. We investigated immunocompetent OBI blood donors from Northern China carrying anti‐HBs as only serological marker of the infection to determine the mechanisms underlying this puzzling condition by examining samples collected prior to the index diagnosis (lookback samples), at index, and during follow‐up. Special attention was paid to the vaccination status of these individuals.

## 2 MATERIALS AND METHODS

### 2.1 Hepatitis B virus serological and molecular testing

Blood donors were pre‐qualified with an HBsAg rapid test (95% limit of detection [LoD]: 5 IU/ml). HBV, HCV and HIV infection status in blood donations collected from HBsAg nonreactive donors was determined according to the serological and molecular screening and confirmatory testing algorithms implemented in the Dalian Blood Center.^16^ HBV DNA quantification was performed with an in‐house real‐time qPCR assay (95% LoD: 20 IU/ml).^17^ Refer to Appendix S1 for further details.

HBV DNA‐positive donations were tested for anti‐HBc with Elecsys® Anti‐HBc II (Roche Diagnostics), Architect® Anti‐HBc II (Abbott Laboratories, Abbott Park) and HISCL Anti‐HBc assay (Sysmex Corporation). Anti‐HBs was tested with Elecsys® Anti‐HBs II (Roche) and HISCL Anti‐HBs assay (Sysmex Corporation), anti‐HBe, and HBeAg with Elecsys® Anti‐HBe and HBeAg (Roche), and anti‐HDV with HDV‐IgG ELISA (WANTAI Bio‐Pharm).

The study was conducted according to the ethical guidelines of the 1975 Declaration of Helsinki and was approved by the Academic and Ethics Committee of Dalian Blood Center (EC‐20190731‐1226). All participants provided signed informed consent at donation time and during follow‐up.

### 2.2 Hepatitis B virus DNA analyses

Hepatitis B virus (HBV) DNA was purified from 6 ml of plasma after polyethylene glycol precipitation of viral particles.^18^ Whole HBV genome, and Precore/Core, Pre‐S/S, S and basic core promoter/pre‐core (BCP/PC) regions were amplified and sequenced as previously described.^17^, ^18^ Sequences were compared with 70 anti‐HBc+/anti‐HBs+ or anti‐HBs− OBI (15 HBV genotype B [HBV_B_] and 51 HBV genotype C [HBV_C_]) and 107 HBsAg+ (58 HBV_B_ and 49 HBV_C_) pre‐qualified donors from Dalian. Phylogenetic analysis was performed as previously described.^17^ Accession numbers from OM471070 to OM471500 were attributed by GenBank to the OBI and non‐OBI sequences from Dalian blood donors included in the study.

### 2.3 Statistical analysis

Categorical variables were compared using Fisher's exact test and continuous variable using the Mann–Whitney test. Multiple group comparison was performed using Kruskal–Wallis non‐parametric test, and interdependence between numerical variables using Spearman rank correlation test. Statistical significance was defined as *p* < .05.

## 3 RESULTS

### 3.1 Hepatitis B virus markers in blood donors

**FIGURE 1:**
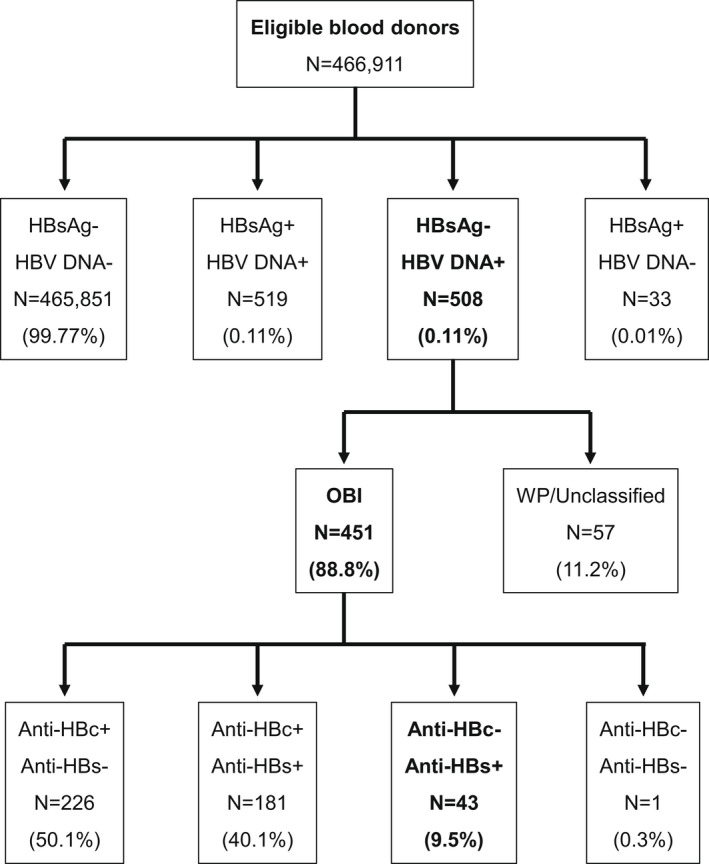
Detection of HBV serological and molecular markers in pre‐qualified blood donors.

From December 2010 to February 2021, 756,971 donations collected from 466,911 pre‐qualified donors were tested for HBsAg by EIAs and for HBV DNA by multiplex HBV/HCV/HIV‐1 nucleic acid testing in either mini‐pools of six plasmas (*n* = 368,352) or, subsequently, in individual donations (*n* = 388,619). HBV infection was confirmed in 1060 (0.22%) qualified donors including 519 (0.11%) HBsAg+/DNA+, 508 (0.11%) HBsAg−/DNA+ and 33 (0.01%) HBsAg+/DNA− (Figure 1). Follow‐up investigations of 476/508 HBsAg−/DNA+ donors (median: 428 days [range: 16–2528]) identified seroconversion to HBsAg and anti‐HBc in 25 (4.9%) HBV DNA‐only reactive donors indicating pre‐seroconversion acute infection, while 451 (88.8%) donors who remained HBsAg negative were classified as OBIs. OBIs included 181 (40.1%) anti‐HBc+/anti‐HBs+, 226 (50.1%) anti‐HBc+/anti‐HBs−, 43 (9.5%) anti‐HBc−/anti‐HBs+ and one (0.3%) seronegative OBI. Lack of anti‐HBc reactivity and anti‐HBs level >10 IU/L were confirmed in isolated anti‐HBs OBIs by three distinct anti‐HBc and two anti‐HBs immunoassays, respectively (Table S1). No follow‐up was available for 32 (6.3%) samples that remained unclassified. All isolated anti‐HBs samples were nonreactive for anti‐HCV, anti‐HIV‐1, anti‐HBc IgM, HBeAg, anti‐HBe and anti‐HDV (data not shown).

### 3.2 Characterization of isolated anti‐HBs OBIs

OBI donors with isolated anti‐HBs were mainly male (60.5%), first‐time donors (67.5%), and ranged in age between 18 and 54 years (median 24 years) (Table 1). They were significantly younger than anti‐HBc+/anti‐HBs+ and anti‐HBc+/anti‐HBs− OBI donors and HBsAg+ donors (*p* < .0001). At index time, all had alanine‐aminotransferase levels <50 IU/ml. The median HBV DNA load of 42 isolated anti‐HBs OBI donors was 20 IU/ml (range: <20–1115 IU/ml), when arbitrarily estimating samples giving no qPCR signal but confirmed DNA positive as containing 5 IU/ml, and qPCR positive samples with signal below the limit of quantification as containing 20 IU/ml. The median viral load was significantly lower in isolated anti‐HBs+ OBIs than in HBsAg+ donors (31 IU/ml [<20–1.45 × 10^8^]), while it was significantly higher than either anti‐HBc+/anti‐HBs+ (5 IU/ml [<20–330]) or anti‐HBc+/anti‐HBs− (5 IU/ml [<20–2570]) OBIs (*p* < .0001). The median anti‐HBs titre was significantly higher in isolated anti‐HBs+ OBI than in anti‐HBc+/anti‐HBs+ OBIs (60.5 IU/L [range: 13–957 IU/L] versus 37 IU/L [range: 10 to >1000 IU/L]; *p* = .0216) (Table 1).

**TABLE 1:** Host and viral markers in HBV‐infected donors

Markers	OBI		Non‐OBI		*p* value
	Anti‐HBc− anti‐HBs+ (*N* = 43)	Anti‐HBc+ anti‐HBs+ (*N* = 181)	Anti‐HBc+ anti‐HBs− (*N* = 226)	HBsAg+^a^ Anti‐HBc+ (*N* = 88)	
Gender (M/F)	26/17	143/38	178/48	50/38	<.0001
Age (years)					
Median	24	45	45	44	<.0001
Range	18–54	19–60	19–60	18–62	
HBV DNA (IU/ml)^b^					
Median	20	5	5	31	<.0001
Range	<20–1115	<20–330	<20–2570	<20–1.45 × 10^8^	
Anti‐HBs (IU/L)^c^					
Median	60.5	37	–	–	.0216
Range	13–957	10 to >1000			
HBV vaccine^d^	31/43 (72%)	11/84 (13%)	4/67 (6%)	2/30 (7%)	<.0001
HBV genotypes^e^					
B	5 (15.2%)	5 (9.8%)	13 (19.4%)	49 (66.2%)	<.0001
C	28 (84.8%)	46 (90.2%)	51 (76.1%)	25 (33.8%)	
D	–	–	3 (4.5%)	–	

^a^ Pre‐qualified donors tested HBsAg positive with EIA.

^b^ HBV DNA load was measured in 42 anti‐HBc−/anti‐HBs+, 81 anti‐HBC+/anti‐HBs+, 102 anti‐HBc+/anti‐HBs− and 87 HBsAg+/anti‐HBc+ samples.

^c^ Average value of anti‐HBs levels measured with two distinct assays.

^d^ Vaccination records only available for 84, 67 and 30 anti‐HBc+/anti‐HBs+ OBIs, anti‐HBc+/anti‐HBs− OBIs and HBsAg+ non‐OBIs, respectively.

^e^ HBV genotyping was available for 33 anti‐HBc−/anti‐HBs+, 51 anti‐HBC+/anti‐HBs+, 67 anti‐HBc+/anti‐HBs− and 74 HBsAg+ samples.

Phylogenetic analysis of whole genome (*n* = 18), PreS/S (*n* = 8), Core (*n* = 7) or partial S (*n* = 6) sequences available for 33 samples identified five (15%) HBV genotype B (HBV_B_) and 28 (85%) genotype C (HBV_C_) sequences. No significant difference in HBV genotype distribution was observed between OBIs irrespective of their serological status with HBV_C_ being dominant. Conversely, HBV_B_ was found dominant in HBsAg+ donors.

Thirty‐one (72%) isolated anti‐HBs OBIs had record of complete HBV vaccination, 17 of whom were vaccinated at birth and 12 between 6 and 16 years of age. Two individuals aged 18 and 21 reported having been vaccinated but could not provide evidence. Of the 11 (26%) unvaccinated individuals, two and seven were 20–21 and 35–54 years old, respectively. No information was available for one donor. Vaccination status collected for 84 anti‐HBc+/anti‐HBs+ OBIs, 67 anti‐HBc+/anti‐HBs− OBIs and 30 HBsAg+ non‐OBIs showed significantly lower vaccination rates of 13%, 6% and 7%, respectively (Table 1). Isolated anti‐HBs OBI subjects were further comparatively stratified between known vaccinated and apparently unvaccinated individuals (Table 2). Vaccinated individuals were younger (22 [18–40] versus 38 [20–54] years; *p* = .0005) and had higher anti‐HBs levels (95.3 [13.5–957] versus 24.5 [13–249] IU/L; *p* = .0011) than unvaccinated individuals. In addition, 48% (15/31) of vaccinated individuals had anti‐HBs >100 IU/L at index. No significant difference in anti‐HBs level was observed between individuals vaccinated at birth and those vaccinated between 6 and 16 years of age (*p* = .69). No significant difference was observed between the two groups regarding HBV DNA load and viral genetic markers (Table 2).

**TABLE 2:** Serological and viral markers in OBI subject with isolated anti‐HBs stratified according to vaccination status

Markers	Vaccinated (*N* = 31)	Unvaccinated (*N* = 11)	*p* value
Gender (M/F)	21/10	5/6	.19
Age (years)			
Median	22	38	.0005
Range	18–40	20–54	
Anti‐HBs (IU/L)			
Median	95	24.5	.0011
Range	13.5–957	13–249	
Anti‐HBs >100 IU/L (N)^a^	15 (48%)	1 (9%)	.0211
Significant anti‐HBs response over time^b^	8/18 (44%)	1/5 (20%)	.61
Detectable anti‐HBs in lookback samples	2/8 (25%)	1/2 (50%)	.49
HBV DNA (IU/ml)			
Median	20	5	.22
Range	<20–1115	<20–215	
HBV genotypes			
B	3 (14%)	2 (33%)	.30
C	18 (86%)	4 (67%)	
S aa substitution rate^c^			
Mean	2.5	1.4	.27
Range	0–11	0–6	
Anti‐HBc conversion	2/15 (13%)	0/5	–

^a^ Average anti‐HBs levels measured at index time.

^b^ A four‐time increase of anti‐HBs titre between two consecutive samples was considered significant.

^c^ 19 and 5 S sequences of vaccinated and non‐vaccinated subjects, respectively, were analysed.

### 3.3 Follow‐up of isolated anti‐HBs OBIs

**FIGURE 2:**
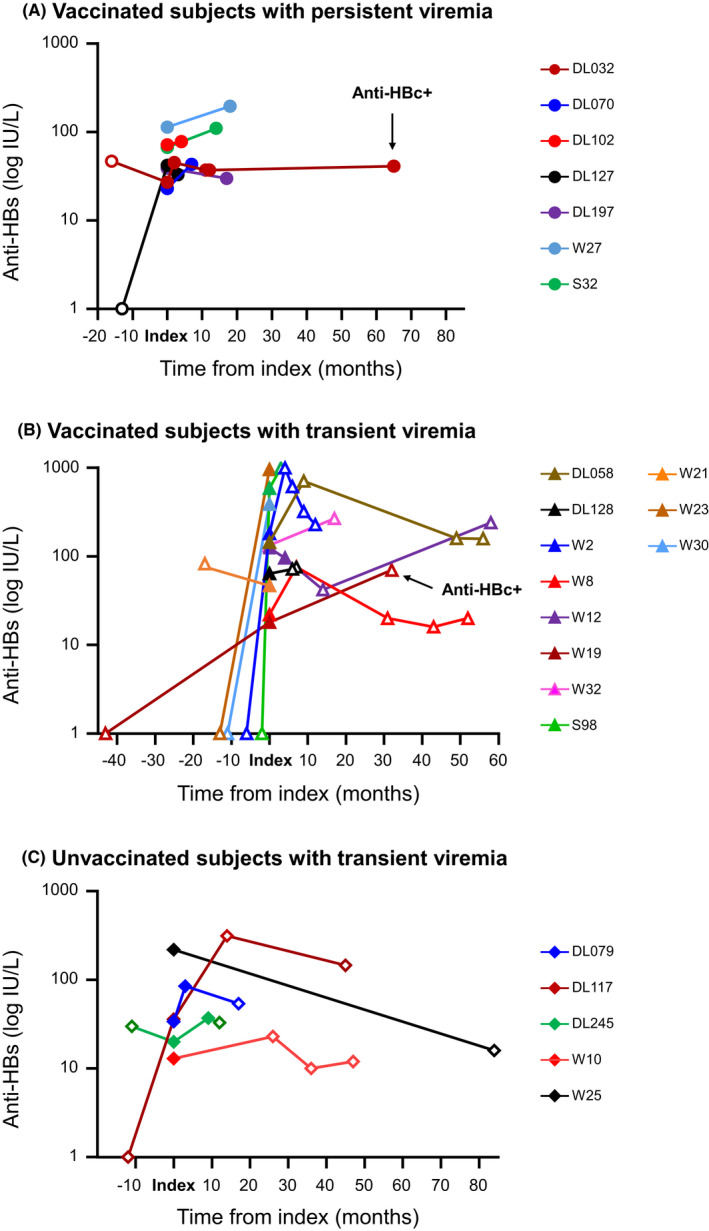
Anti‐HBs and HBV DNA detection over time in vaccinated and unvaccinated isolated anti‐HBs OBI carriers. Open symbols indicate undetectable HBV DNA. Arrows indicate anti‐HBc positive samples.

Twenty (46.5%) isolated anti‐HBs OBIs, including 15 vaccinated and five unvaccinated subjects, returned 1–4 times for follow‐up over an average period of 27.1 months (range: 2.6–84.3 months). All but two subjects consistently tested negative for anti‐HBc (Table S1). Vaccinated subjects DL032 and W19 seroconverted to anti‐HBc sometime between 11.6–65.1 months and 31.5 months after index donation, respectively. HBV DNA was consistently detected in seven vaccinated subjects, including DL032, by at least one of the nucleic acid testing assays used over a follow‐up of 2.6–65.1 months (Figure 2A). HBV DNA was reactive in samples from two vaccinated and two unvaccinated individuals collected 2.5–8.7 months after index collection but became undetectable when tested 12.2–57.9 months later (Figure 2B,C). HBV DNA was no longer detected in samples collected 3.7–84.3 months post index in six vaccinated, including W19, and three unvaccinated individuals (Figure 2B,C). There was no relationship between the follow‐up outcome of viraemia and HBV genotypes. At index time, anti‐HBs level was higher in vaccinated individuals who became nonviraemic (median: 129.5 [17.5–586.5] IU/L) compared to those consistently viraemic (median: 42 [22.5–113.5] IU/L) and to nonviraemic unvaccinated individuals (median: 34 [13–249] IU/L), but the difference was not statistically significant.

Lookback samples of eight vaccinated and two unvaccinated repeat donors collected 2.4–16.6 months before the viraemic index donation were available. No HBV marker was detected in any of these samples except anti‐HBs in vaccinated DL032 (47 IU/L) and W21 (81 IU/L), and unvaccinated DL245 (30 IU/L) (Table S1 and Figure 2). Using index anti‐HBs value as baseline, the change over time in anti‐HBs level was analysed in 18 vaccinated and five unvaccinated individuals. Arbitrarily considering that a fourfold or greater increase in anti‐HBs titre indicates a significant anti‐HBs response, an anti‐HBs response was observed in 44% (8/18) and 20% (1/5) of vaccinated and unvaccinated individuals, respectively, and was associated with transient HBV viraemia in all cases except DL127 (Table 2 and Figure 2A).

### 3.4 Genetic analysis of anti‐HBs only OBI sequences

The BCP/PC sequences of four isolated anti‐HBs OBI genotype B (OBI_B_) strains and 25 anti‐HBc−/anti‐HBs+ OBI genotype C (OBI_C_) strains were analysed together with the sequences of 35 anti‐HBc+/anti‐HBs+ OBI (3 HBV_B_ and 32 HBV_C_) and with 34 anti‐HBc+/anti‐HBs− OBI (8 HBV_B_ and 26 HBV_C_) donors from Dalian, China (Figure S1). No major differences were observed in the OBI sequences stratified according to donor serostatus. No specific mutation was identified in the HBV X sequences available for 18 isolated anti‐HBs OBIs (Figure S5).

Core amino acid (aa) sequences of four isolated anti‐HBs OBI_B_ and 22 OBI_C_ were aligned with the corresponding consensus sequences derived from 25 HBV_B_ and 14 HBV_C_ sequences of HBsAg+ donors. All nucleotide substitutions observed in isolated anti‐HBs OBI_C_ sequences were synonymous substitutions, except a V91I substitution in the Core protein of sample W2 (Figure S2). A significantly higher aa variability was observed in 23 anti‐HBc+/anti‐HBs+ OBI_C_, 19 anti‐HBc+/anti‐HBs− OBI_C_ and 14 HBsAg+ HBV_C_ sequences (Table S2). The reverse was observed between isolated anti‐HBs and anti‐HBc+ OBI_B_ sequences but this may be related to the limited number of OBI_B_ sequences available and the presence of nine aa substitutions and one aa deletion in sample W33 sequence (Figure S2).

Pre‐S1, Pre‐S2 and/or S aa sequences of 3 isolated anti‐HBs OBI_B_ and 22 isolated anti‐HBs OBI_C_ strains were compared to their respective anti‐HBc+ OBIs and HBsAg+ counterparts (Table S2). The average aa diversity over the long surface protein was significantly lower in isolated anti‐HBs OBI strains than in both anti‐HBc+ OBI and HBsAg+ strains irrespective of HBV genotype (*p* < .0001). In particular, the major hydrophilic region (MHR) sequence of 20 (91%) isolated anti‐HBs OBI_C_ strains was identical to the corresponding HBsAg+ consensus sequence. Moreover, the entire S protein sequences of 14 (64%) isolated anti‐HBs OBI_C_ were identical to the consensus HBV_C_ sequence (Figures S3 and S4). To rule out possible cross‐contaminations, the whole viral DNA analysis process was repeated at least twice at different times in different settings using stored plasmas, and identical sequencing results were obtained (data not shown). Still, multiple aa substitutions were present in some isolated anti‐HBs OBI_B_ and OBI_C_ S proteins, including 54 substitutions not found in HBsAg+ controls, of which 23 (43%) were present in anti‐HBc+ OBI strains, although mainly in only single OBI strain (Table 3). Few substitutions occurred at sites previously identified to be critical for antigenicity and/or immune escape, including sT116S, sT118K, sP120, sG130N, sT131K, sP142L, sG145R, sC149Y, sA166V and sI195R. In addition, samples DL245 and S99 had a serine residue insertion at position s115 and a 4‐aa deletion at positions s117‐s120, respectively.

**TABLE 3:** Amino acid substitutions specifically observed in the HBV envelop proteins of anti‐HBc−/anti‐HBs+ OBI strains

Sample ID	PreS1	PreS2	S
OBI_B_			
DL070			S31N, **M57I**, **T131K** ^**a**^, **C149Y** ^**a**^, **R169C**
DL245^b^			**P111T**, **115S**, **P142L** ^**a**^, G145R^a^, **E164D**, **A166V** ^**a**^, **F170S**
W33	**F34L**, **N51I**		**I81T**
OBI_C_			
DL032	T14A		T118K^a^
DL035	**G35E**	**F46Y**	
DL058	**P94T**		**I25L**
DL102			S6L, **V96G**, T116S^a^, K122R, P127S, L216F
S99			L109R, L110R, **S117Δ**, **T118Δ** ^**a**^, **G119Δ**, **P120Δ** ^**a**^, **G130N** ^**a**^, S136Y, A159V
W10^b^		**T7A**	
W12			S210N
W13	**T87A**		
W18			K122R, P217L, **L222P**, I226T
W19	H51R, T87I		
W22		**S40L**	S114T, T131P
W24^b^	N56K, **P93S**		T118K^a^, **V180A**, **I195R** ^**a**^
W27	**Q100K**		
W34	**A90I**		

*Note*: Amino acid substitutions indicated in bold were found uniquely in anti‐HBc negative OBI strains; other substitutions were also present in 15 and 55 anti‐HBc positive OBI_B_ and OBI_C_ strains, respectively. Position numbering is according to the first methionine of each large (PreS1), medium (PreS2) and small (S) surface protein.

^a^ Substitutions occurring at positions previously reported associated with altered antigenicity and/or immune escape.

^b^ Unvaccinated individuals.

**FIGURE 3:**
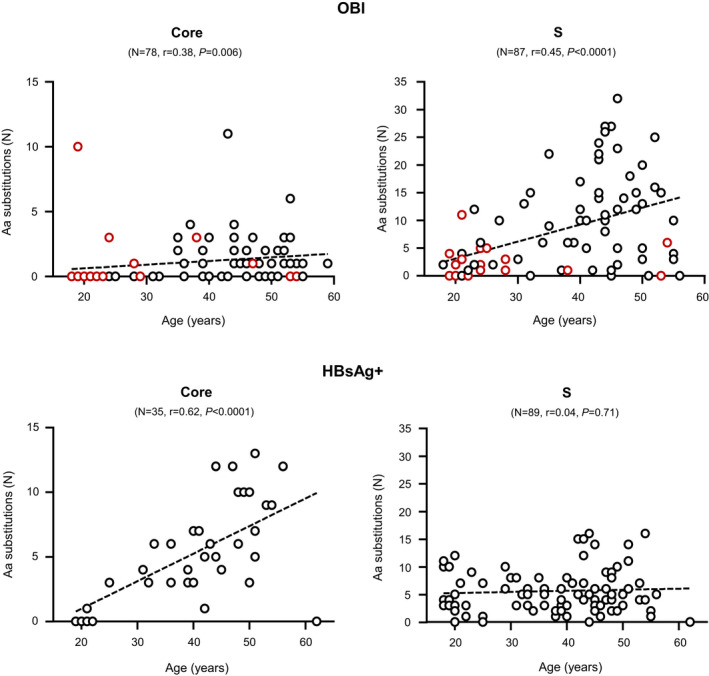
Correlation between age of OBI and HBsAg+ carriers and the level of amino acid mutation in viral Core and S proteins. Dots represent individual values. Red dots indicate isolated anti‐HBs OBIs. Spearman rank correlation test was used.

Amino acid substitution rate in the Core and the small S proteins was analysed in OBI, irrespective of serostatus, and HBsAg+ individuals stratified according to age as a surrogate for duration of infection (Figure 3). In OBI carriers, a relatively small but significant positive correlation was observed between age and the number of substitutions in Core (Spearman *r* = .38 [95% CI: 0.17–0.56]; *p* = .0006) and especially in S (Spearman *r* = .45 [95% CI: 0.26–0.61]; *p* < .0001) (Figure 3). As shown in Table 2, The mean S aa substitution rate was not significantly different between vaccinated and unvaccinated anti‐HBc−/anti‐HBs+ OBIs (2.5 [0–11] versus 1.4 [0–6] residues; *p* = .27). In contrast, HBsAg+ individuals showed a positive correlation between age and aa substitutions in the Core (Spearman *r* = .62 [95% CI: 0.36–0.80]; *p* < .0001) but not in the S protein.

## 4 DISCUSSION

Occult hepatitis B infection was identified as a distinct feature in HBV infection natural history and can be associated with unusual viral marker profiles that add complexity to its diagnosis in asymptomatic carriers. Between 2010 and 2021, the prevalence of confirmed OBI in blood donors from Dalian, China, was 1:1035, consistent with frequencies approximating 1:1000–3000 previously reported in China and South East Asia where HBV genotypes B and C are dominant.^8^, ^19^, ^20^ From an overall 451 donors with confirmed OBI, half carried anti‐HBc only, 40.1% both anti‐HBc and anti‐HBs, 9.5% isolated anti‐HBs, and 0.3% no serological marker (Figure 1). This distribution was similar to previous reports from China and South East Asia.^2^, ^8^, ^9^ The present study focused on the selected group of 43 OBI blood donors with anti‐HBs as isolated serological marker of infection.

Absence or delayed anti‐HBc reactivity in patients with overt HBV infection has been associated with immunosuppression, HIV co‐infection, insufficient sensitivity of anti‐HBc assays and rare HBcAg variants.^3^, ^4^, ^5^, ^6^ None of these conditions applied to the present OBI cases. All isolated anti‐HBs OBI donors were apparently immunocompetent with no evidence of liver disease, medical treatment or known viral co‐infection. Anti‐HBc false negative result was excluded by using three distinct assays, one of which included antigen–antibody complex dissociation pre‐treatment. No specific mutations in BCP/PC, Core and HBx that could alter HBcAg production or anti‐HBc detection were identified, consistent with studies of overt anti‐HBc‐negative HBV infection.^6^, ^21^

The minority OBI group with isolated anti‐HBs differed from the other two OBI groups by a significantly larger percentage of females, younger age, higher (although still relatively low) levels of viral DNA and anti‐HBs, and higher frequency of HBV vaccination (Table 1). The 72% vaccination rate observed in isolated anti‐HBs OBIs was consistent with the age distribution (median: 24 years [range: 18–54 years]) and the initiation of the Chinese universal HBV vaccination program in 1992. Isolated anti‐HBs OBIs were therefore stratified between 31 vaccinated and 11 unvaccinated with 87% of the former younger than 30 years and 73% of the latter group older than 30 (Tables S1 and Table 2). In addition to age, the 31 vaccinated isolated anti‐HBs OBIs differed in several other aspects: mostly males (68%), higher level of anti‐HBs at index, and more frequent, albeit not significantly, HBV genotype C (Table 2). However, both groups had in common an absence of DNA or anti‐HBc in lookback samples, persistence of isolated anti‐HBs status in follow‐up, and absence or paucity of amino acid substitutions in the Core and S MHR, respectively (Table S1, Figures S2 and S4, and Table 3). Due to the vaccination history of the majority of individuals with isolated anti‐HBs OBI, three main hypotheses were examined to explain the mechanisms underlying this condition: (i) long‐term persistence of OBI status acquired in infancy naturally or from infectious mother or relatives concomitantly or soon after vaccination, (ii) subclinical vaccine breakthrough infection with an HBV escape variant and (iii) unconventional infection acquired post‐vaccination.

Isolated anti‐HBs OBI has been reported in vaccinated infants aged 6–98 months born to chronically infected mothers.^10^, ^11^, ^12^, ^14^, ^22^ HBV DNA levels were consistently low, but often only transiently persistent beyond 1–4 years of age when follow‐up was available.^11^, ^13^, ^23^ A Taiwanese cross‐sectional study reported a prevalence of HBV viraemia of about 3%–5% in anti‐HBc−/anti‐HBs+ children aged <6 to 18 years, with a peak prevalence of ~12% in children 6–10 years old, and approximately 4% beyond 18 years of age.^10^ In the present study, the HBV infection status of the corresponding mothers or relatives was not known, except for subject W30, a 19‐year‐old female born to mother with chronic HBV infection. This option appears unlikely in both vaccinated and unvaccinated isolated anti‐HBs OBI donors since none of the available lookback samples preceding index samples by an average of 1.2 years was DNA positive, and 65% (13/20) of follow‐up samples became DNA negative (Table S1 and Figure 2). However, low level of HBV DNA fluctuating around the LoDs of assays has been observed in both anti‐HBc+ and isolated anti‐HBs OBIs sometimes over several years.^8^, ^24^, ^25^ Therefore, long‐term OBI carriage with intermittently detectable viraemia over a long period of time in fully immunized subjects cannot be totally ruled out but remains improbable.

While the effectiveness of vaccination programs in reducing the prevalence of CHB is well established, their impact on the prevalence of HBV immune escape variants remains unclear.^26^ Mutations associated with evasion of vaccine‐induced immunity have been localized frequently, but not exclusively, in the MHR of the viral surface protein.^27^ However, 70% (23 [2 OBI_B_ + 21 OBI_C_]/33) of the isolated anti‐HBs OBI strains had wild‐type MHR sequences. Only four vaccinated and two unvaccinated strains carried 1–3 mutations previously associated with immune escape as indicated in Table 3. Similarly, few large studies reported wild‐type MHR sequences in 75%–83% of vaccinated isolated anti‐HBs OBI carriers infected with HBV_B_ and HBV_C_.^26^, ^28^, ^29^ Nevertheless, multiple substitutions were also present outside the MHR of single isolated anti‐HBs OBI strains including several not found in anti‐HBc+ OBI and non‐OBI strains (Table 3). However, it would be highly speculative to associate these substitutions with impaired anti‐HBs neutralization without conducting appropriate functional investigations.

Long‐term OBI carriage and breakthrough infection with vaccination‐related viral variants being unlikely, isolated anti‐HBs OBI in vaccinated adults appeared to reflect recent infection with wild‐type viruses for several reasons. First, nearly half (15/31) of the index samples had anti‐HBs levels greater than 100 IU/L, which is well above what would be expected 10–20 years after teenage or at birth HBV vaccination, respectively. Such levels may suggest relatively recent contact with HBV boosting anti‐HBs immunity. This was further supported by anti‐HBs being undetected in six of eight (75%) lookback samples from vaccinated donors (Figure 2). In addition, eight of 18 (44%) vaccinated and one of five (20%) unvaccinated individuals with lookback and/or follow‐up samples showed four or more times increase of anti‐HBs level, four of them after <6 months suggesting an immune response to recent HBV contact (Table 2 and Figure 2). Further, vaccinated isolated anti‐HBs OBI presented with higher, although not significantly, HBV DNA levels than those unvaccinated, compatible with recent contact with HBV. Finally, 2 of 20 (10%) individuals with follow‐up seroconverted to anti‐HBc. In the present study, the source and the timing of potential HBV exposure were unknown. However, isolated anti‐HBs OBIs showed a significantly lower frequency of substitutions in both Core and S than anti‐HBc+ OBIs (Table S2), which may suggest a shorter infection history. Considering that HBV infection occurs mainly at an early age in Asia, and despite the low level of HBV replication in OBI irrespective of anti‐HBc status, mutations seem to accumulate over time in OBI virus genome as suggested in Figure 3 when the age of infected individuals was used as a surrogate for the duration of infection. Overall, several lines of evidence support that vaccinated young blood donors presenting with isolated anti‐HBs OBI result of relatively recent exposure to HBV, possibly through sexual contact, evidenced by the occurrence of mostly low‐level HBV DNA and boosting an anti‐HBs response, rarely accompanied by a long‐delayed anti‐HBc response.

Follow‐up showed that seven of 15 (47%) vaccinated individuals remained HBV DNA low but detectable two to 65 months post index sample, while 8/15 (53%) became HBV DNA negative (Figure 2). All five unvaccinated individuals follow up had undetectable DNA 8 months post index sample at the latest. Overall, nine of 10 individuals with follow‐up samples keeping stable anti‐HBs levels remained HBV DNA positive two to 65 months post index sample while 6/7 donors with anti‐HBs response became HBV DNA negative, suggesting efficacy of the boosted anti‐HBs response. The transient viraemia observed in 65% (13/20) of isolated anti‐HBs OBI carriers suggests ‘aborted’ infection that is reminiscent of acute HBV infection previously reported in six vaccinated blood donors who carried isolated anti‐HBs OBI; four of them infected by high viral load HBV sexual partners.^15^ Differing from the cases described here, three of four cases with detailed follow‐up developed delayed low‐level anti‐HBc 1–4.5 months post index, while one who was infected with HBV genotype B showed no anti‐HBc reactivity over 9 months follow‐up. In addition, none of these individuals showed persistent viraemia after 135 days of follow‐up.^15^ These differences between the two studies may be due to the infected individuals in the Stramer study having been vaccinated as adults and had been exposed to different HBV genotypes and high viral loads. HBV infection in vaccinated individuals could induce faster innate and adaptive immune responses that may limit and possibly suppress viral replication in the presence of pre‐existing low level of anti‐HBs.^30^ A rapid suppression of low viraemia may limit the production of HBcAg and prevent the development of a specific immune response. It is possible that the paucity and often long delays of follow‐up sample collection in the present study missed low‐level anti‐HBc and transient viraemia. Hepadnaviral persistence in the absence of detectable serum surface antigen and antibodies to Core has been described in animals inoculated with low dose of woodchuck hepatitis virus and offspring of woodchuck dam convalescent from acute infection.^31^ Similarly, exposure of vaccinated subjects to a small amount of virus in the presence of non‐sterilizing anti‐HBs might result in isolated anti‐HBs OBI. The persistence of the isolated anti‐HBs OBI profile observed in 11 unvaccinated donors in the present study and in 9 Southeast Asian donors in a previous study remains puzzling.^8^ It is possible that some of these donors, particularly the two younger than 30, might have been vaccinated without recollection or evidence provided. Further studies are needed to determine whether this inability to produce anti‐HBc might be related to a selective defect in the host immune system.^6^, ^32^ Overall, the prevalence of isolated anti‐HBs OBI carriage may be underestimated in vaccinated populations, especially in areas of high HBV endemicity, and these individuals may be at risk for HBV reactivation if immunosuppressed. In addition, HBsAg‐negative/anti‐HBc‐negative blood donors with barely detectable HBV DNA may participate to the residual risk of HBV transfusion‐transmission, although this risk is likely mitigated by the presence of anti‐HBs.^33^

In conclusion, occurrence and persistence over time in rare cases of isolated anti‐HBs OBI were confirmed in Chinese blood donors. Isolated anti‐HBs OBI appears mainly associated with vaccinated adult blood donors exposed to HBV one‐ or two‐decades post‐vaccination who remain largely protected from full blown HBV breakthrough infections and develop low level aborted infection revealed by transient HBV DNA and an immune anti‐HBs response. However, a subset of individuals still experienced low but persistent viraemia and the potential for transmission and long‐term clinical outcome of this uncommon OBI condition remain uncertain.

## CONFLICT OF INTEREST

The authors declare no conflict of interest.

## Supporting information


Appendix S1

## Data Availability

The data that support the findings of this study are openly available in GenBank at https://www.ncbi.nlm.nih.gov/nucleotide/, reference number OM471070‐OM471500.
