# Supplementary material for: Anti‐HBc‐nonreactive occult hepatitis B infections with HBV genotypes B and C in vaccinated immunocompetent adults
Source: J Viral Hepat. 2022 Aug 24;29(11):958–67. doi: 10.1111/jvh.13733 (PMC9804389; doi:10.1111/jvh.13733)
Supplement: Supplementary file 1 — Appendix S1 [file JVH-29-958-s001.docx]

**Supplementary materials**

**Anti-HBc-nonreactive occult hepatitis B infection associated with HBV genotypes B and C infections in vaccinated individuals**

Xuelian Deng, Xiaohan Guo, Hongfang Gu, Dong Wang, Syria Laperche, Jean-Pierre Allain, Liang Zang, Daniel Candotti

Table of contents

Supplementary materials and methods…………………………………………………….2

Supplementary Table S1…………………………………………………………………….4

Supplementary Table S2……………………………………………………………………12

Supplementary figures………………………………………………………………………13

Figure S1…….…….………………………………….………………………………13

Figure S2………….……………………………………..……………………………16

Figure S3…….…….………………………………….………………………………21

Figure S4………………………………………………………………………………27

Figure S5………………………………………………………………………………35

**Supplementary materials and methods**

**Serological and molecular screening of blood donations**

After candidate blood donors completed a questionnaire to assess their general health and risky and physical examination was conducted by a dedicated clinician, a blood sample (2 mL) was collected for pre-donation testing that included blood type, hemoglobin (exclusion thresholds: <115 [female] - <120 [male] g/mL), alanine-aminotransferase (ALT) levels (exclusion thresholds: >50 IU/mL), and HBsAg using a rapid test (HBsAg Rapid test, In Tec Products, Xiamen, China; limit of detection [LoD]: 5 IU/mL).

According to Chinese regulations, all blood donations are mandatorily screened with two enzymatic immunoassays (EIAs) simultaneously for HBsAg, HCV antibodies, HIV antibodies alone or in combination with HIV antigen, and antibodies to Treponema pallidum. For each serological marker tested, samples that were independently reactive with a given assay were re-tested twice with the same assay; at least one reactive result qualified the sample as initially reactive (IR). Donations that were IR for HBsAg were tested further with an electro-chemiluminescent assay. Details of the assays are provided in Deng *et al*. Transfusion 2020;60:334-342.

Viral nucleic acid testing (NAT) for HBV DNA, HCV RNA, and HIV RNA was performed alternatively using two different multiplex assays to reduce the risk of reagent shortage. Donations were tested randomly with either assay based on reagent availability. The cobas® TaqScreen MPX 1.0 Test (95% LoDs: HBV 3.8 IU/mL, HCV 11 IU/mL, and HIV-1 49 IU/mL) and the cobas® TaqScreen MPX Test V2.0 (95% LoDs: HBV 2.3 IU/mL, HCV 6.8 IU/mL, and HIV-1 50.3 IU/mL, Roche Diagnostics) were used in mini-pools of six plasmas samples from 2010-2014 and 2015-2021, respectively. IR pools were resolved by testing each individual sample with the same assay. Alternatively, NAT was done in individual donations (ID) by using the PROCLEIX ULTRIO® assay (95% LoDs: HBV 10.4 IU/mL, HCV 3 IU/mL, and HIV-1 29 IU/mL [Novartis, San Diego, USA]) between 2011-2015 and the PROCLEIX ULTRIO® Plus or Procleix Ultrio^®^ Elite (95% LoDs: HBV 3.4 IU/mL, HCV 5.4 IU/mL, and HIV-1 21.2 IU/mL [Grifols, Barcelona, Spain]) between 2016 and 2021. Reactive samples were discriminated by using the corresponding discriminatory assays. Discriminated NAT reactive samples with repeat reactive serology results were considered definitively reactive and were not investigated further. NAT reactivity was confirmed only in seronegative samples by individual testing of the plasma unit. Donations that were NAT IR were tested once with the COBAS Ampliprep/COBAS TaqMan assays or twice with Ultrio or Ultrio Plus, according to reagent availability. One reactive result classified the sample as definitively reactive.

**Sequence diversity analysis**

Intra-group sequence diversity was defined as the mean value for pairwise distance between sequences within the same group, calculated as the number of nucleotide or amino acid (aa) differences between two individual sequences, corrected for sequence length. Progressive accession numbers from OM471070 to OM471500 were attributed by GenBank to the OBI and non-OBI sequences from Dalian blood donors included in the study.

**Supplementary Table S1**

Table S1. Characteristics of 43 anti-HBs only OBIs at index time and follow-up.

| ID | Gender | Age  (years) | Donor type | Vaccine  (age) | Time from index (days) | HBV DNA | Viral load  (IU/mL) | HBsAg | Anti-HBs  assays  1/2^†^  (IU/L) | Anti-HBc  assays  1/2/3^‡^ | HBV  genotype |
| --- | --- | --- | --- | --- | --- | --- | --- | --- | --- | --- | --- |
| DL032 | M | 25 | Repeat | Yes (6-12y) | -488  Index  57  330  349  1,953 | -  **+**  **+**  **+**  **+**  **+** | Undetected  **<20**  Undetected | -  -  -  -  -  - | **47**/NT  **37/17**  **45**/NT  **37**/NT  **37**/NT  **41**/NT | -/NT/NT  -/-/-  -/-/-  -/-/-  -/-/-  **+/+/+** | C |
| DL035 | M | 18 | 1^st^ time | Yes | Index | **+** | **<20** | - | **441/341** | -/-/- | C |
| DL058 | M | 21 | 1^st^ time | Yes (6-12y) | Index  268  1,475  1,677 | **+**  -  -  - | **<20**  Undetected | -  -  -  - | **164/123**  **711**/NT  **160**/NT  **159**/NT | -/-/-  -/-/-  -/-/-  -/-/- | C |
| DL070 | M | 24 | 1^st^ time | Yes (6-12y) | Index  199 | **+**  **+** | Undetected | -  - | **24/21**  **43**/NT | -/-/-  -/-/- | B |
| DL079 | M | 36 | 1^st^ time | No | Index  101  523 | **+**  **+**  - | Undetected  **<20** | -  -  - | **40/28**  **85**/NT  **54**/NT | -/-/-  -/-/-  -/-/- | NA |
| DL102 | M | 21 | 1^st^ time | Yes | Index  108 | **+**  **+** | **155** | -  - | **62/82**  **78**/NT | -/-/-  -/-/- | C |
| DL117 | M | 38 | Repeat | No | -358  Index  41  224  1,355 | -  **+**  -  -  - | Undetected  Undetected | -  -  -  -  - | -/NT  **36**/NT  **314**/NT  **455**/NT  **146**/NT | -/NT/NT  -/-/-  -/-/-  -/NT/NT  -/-/- | B |
| DL127 | F | 21 | Repeat | Yes (16 y) | -381  Index  77 | -  **+**  **+** | **<20** | -  -  - | -/NT  **53/31**  **33**/NT | -/NT/NT  -/-/-  -/-/- | C |
| DL128 | M | 21 | 1^st^ time | Yes (birth) | Index  184 | **+**  - | Undetected | -  - | **64**/NT  **72**/NT | -/-/-  -/-/- | NA |
| DL197 | M | 19 | 1^st^ time | Yes (birth) | Index  513 | **+**  **+** | NA  Undetected | -  - | **43/35**  **30**/NT | -/-/-  -/-/- | NA |
| DL243 | F | 22 | Repeat | Yes (birth) | Index  180 | **+**  **+** | **91**  **<20** | -  - | **-/-**  **138/151** | -/-/-  -/-/NT | C |
| DL245 | F | 48 | Repeat | No | -335  Index  261  366 | -  **+**  **+**  - | **215**  Undetected  Undetected | -  - - - | **30**/NT  **22/18**  **52/22**  **33/33** | -/NT/NT  -/-/-  -/-/-  -/-/- | B |
| W2 | M | 28 | Repeat | Yes (6-12y) | -193  Index  114  179  268  360 | -  **+**  -  -  -  - | **28**  Undetected | -  -  -  -  -  - | -/NT  **190/170**  **1,000/1,000**  **616**/NT  **320**/NT  **228**/NT | -/-/-  -/-/-  -/-/-  -/NT/NT  -/NT/NT  -/-/- | C |
| W8 | F | 20 | 1^st^ time | Yes (birth) | Index  214  942  1,304  1,548 | -  **+**  -  -  - | **<20**  Undetected  Undetected | -  -  -  -  - | **27/16**  **76**/NT  **25/15**  **16**/NT  **20**/NT | -/-/-  -/-/-  -/-/-  -/-/-  -/-/- | C |
| W10 | F | 20 | 1^st^ time | No | Index  767  1,094  1,409 | **+**  NT  -  - | **<20**  Undetected  Undetected | -  -  -  - | **16/10**  **30/16**  **10**/NT  **12**/NT | -/-/-  -/-/-  -/-/-  -/-/- | C |
| W12 | M | 20 | 1^st^ time | Yes (birth) | Index  109  432  1,737 | **+**  **+**  -  - | **<20**  Undetected | -  -  -  - | **110/140**  **96/96**  **42**/NT  **264/215** | -/-/-  -/-/-  -/-/-  -/-/- | C |
| W13 | F | 20 | 1^st^ time | NA | Index | **+** | Undetected | - | **53/25** | -/-/- | C |
| W14 | F | 21 | 1^st^ time | No | Index | **+** | Undetected | - | **27/13** | -/-/- | C |
| W15 | F | 53 | Repeat | No | Index | **+** | **<20** | - | **21/16** | -/-/- | C |
| W16 | M | 33 | 1^st^ time | Yes (13-15y) | Index | **+** | **<20** | - | **15/12** | -/-/- | C |
| W17 | F | 24 | 1^st^ time | Yes (birth) | Index | **+** | **20** | - | **67/40** | -/-/- | C |
| W18 | M | 25 | Repeat | Yes (6-12y) | Index | **+** | **<20** | - | **749/450** | -/-/- | C |
| W19 | M | 22 | Repeat | Yes (birth) | -1,281  Index  946 | -  **+**  - | **<20** | -  -  - | -  **20/15**  **70**/NT | -/-/-  -/-/-  **+**/NT/NT | C |
| W21 | F | 29 | Repeat | Yes (13-15y) | -497  Index | -  **+** | Undetected | -  - | **81**/NT  **34/60** | -/-/-  -/-/- | NA |
| W22 | F | 20 | 1^st^ time | Yes (birth) | Index | **+** | Undetected | - | **594/375** | -/-/- | C |
| W23 | M | 19 | Repeat | Yes (birth) | -386  Index | -  **+** | **32** | -  - | -/NT  **914/>1,000** | -/-/-  -/-/- | C |
| W24 | F | 54 | 1^st^ time | No | Index | **+** | Undetected | - | **20/53** | -/-/- | C |
| W25 | M | 26 | 1^st^ time | No | Index  2,530 | **+**  - | Undetected  Undetected | -  - | **210/228**  **16**/NT | -/-/-  -/NT/NT | NA |
| W26 | M | 22 | 1^st^ time | Yes (birth) | Index | **+** | **182** | - | **43/34** | -/-/- | C |
| W27 | F | 19 | 1^st^ time | Yes (birth) | Index  534 | **+**  **+** | Undetected  Undetected | -  - | **148/79**  **196**/NT | -/-/-  -/-/NT | C |
| W28 | F | 24 | 1^st^ time | Yes (birth) | Index | **+** | **1,115** | - | **788/345** | -/-/- | B |
| W29 | F | 35 | 1^st^ time | No | Index | **+** | **<20** | - | **14/16** | -/-/- | NA |
| W30 | F | 28 | Repeat | Yes (birth) | -324  Index | -  **+** | **<20** | -  - | -/-  **623/150** | -/-/-  -/-/- | C |
| W31 | M | 40 | 1^st^ time | Yes (13-15y) | Index | **+** | Undetected | - | **153/218** | -/-/- | C |
| W32 | M | 32 | 1^st^ time | Yes (6-12y) | Index  512 | **+**  - | Undetected  Undetected | -  - | **134/134**  **269**/NT | -/-/-  -/NT/NT | NA |
| W33 | M | 19 | 1^st^ time | Yes (birth) | Index | **+** | **358** | - | **87/123** | -/-/- | B |
| W34 | M | 24 | 1^st^ time | Yes (birth) | Index | **+** | **<20** | - | **29/27** | -/-/- | C |
| W35 | F | 29 | 1^st^ time | Yes (6-12y) | Index | **+** | Undetected | - | **96/77** | -/-/- | NA |
| W36 | M | 20 | 1^st^ time | Yes (birth) | Index | **+** | Undetected | - | **116/92** | -/-/- | NA |
| W37 | M | 47 | Repeat | No | Index | **+** | Undetected | - | **21/28** | -/-/- | NA |
| S32 | M | 33 | 1^st^ time | Yes (6-12y) | Index  413 | **+**  **+** | Undetected  **279** | -  - | **83/51**  **110**/NT | -/-/NT  -/NT/NT | C |
| S98 | M | 22 | Platelets  apheresis | Yes (birth) | -71  Index  77 | -  **+**  - | Undetected  Undetected | -  -  - | -/NT  **739/434**  **1,000**/NT | -/NT/NT  -/-/NT  -/NT/NT | C |
| S99 | M | 40 | 1^st^ time | No | Index | **+** | Undetected | - | **50/27** | -/-/NT | C |

^†^Assay 1, Elecsys Anti-HBs II; assay 2, HISCL Anti-HBs.

^‡^Assay 1, Elecsys Anti-HBc II; assay 2, Architect Anti-HBc II; assay 3, HISCL Anti-HBc.

NA, not available; NT, not tested.

**Supplementary table S2**

Table S2. Average intra-group amino acid diversity of HBV genotypes B and C sequences^†^.

| HBV proteins |  | | Genotype B | | | | |  | | Genotype C | | | | | |  |
| --- | --- | --- | --- | --- | --- | --- | --- | --- | --- | --- | --- | --- | --- | --- | --- | --- |
|  |  | OBI  anti-HBc-  anti-HBs+ | | OBI  anti-HBc+  anti-HBs+ | OBI  anti-HBc+  anti-HBs- | Non-OBI  HBsAg+  anti-HBc+ | *P* value^‡^ | |  | | OBI  anti-HBc-  anti-HBs+ | OBI  anti-HBc+  anti-HBs+ | OBI  anti-HBc+  anti-HBs- | Non-OBI  HBsAg+  anti-HBc+ | *P* value^‡^ | |
| Core  N  Mean  Range |  | 4  5.2  2.4-7.3 | | 2  0.5  NA | 6  2.3  0.0-4.4 | 25  5.7  0.0-10.4 | <0.0001 | |  | | 22  0.05  0.0-0.6 | 23  1.6  0.0-8.3 | 19  1.2  0.0-3.9 | 14  3.3  0.0-9.3 | <0.0001 | |
| PreS1/S2  N  Mean  Range |  | 3  3.6  2.6-5.3 | | - | 6  11.7  5.7-21.9 | 58  4.3  0.0-13.8 | <0.0001 | |  | | 22  1.8  0.0-4.0 | 24  4.3  0.0-9.8 | 27  5.4  1.2-11.6 | 49  3.9  0.0-10.9 | <0.0001 | |
| S  N  Mean  Range |  | 3  2.8  2.2-3.5 | | 1  NA | 10  11.3  8.9-21.2 | 58  5.1  0.4-13.3 | <0.0001 | |  | | 22  1.6  0.0-7.1 | 24  7.1  0.0-15.7 | 27  8.3  0.0-17.8 | 49  3.4  0.0-9.4 | <0.0001 | |

^†^S partial sequences were not considered in the analysis.

^‡^Kruskal-Wallis test used.

NA, not available.

**Supplementary figures**

**A**

**B**

**C**

**Supplementary figure S1. Alignment of HBV BCP/PC nucleotide sequences.** Sequences of anti-HBc-/anti-HBs+ OBIs (A), anti-HBc+/anti-HBs+ OBIs (B), and anti-HBc+/anti-HBs- OBIs (C) were aligned with consensus sequences derived from 64 HBsAg+ genotype B sequences and 50 genotype C sequences from asymptomatic blood donors. HBV_B_ and HBV_C_ consensus sequences were identical. Nucleotides identical to the reference consensus are indicated by dots.

**A**

**B**

**C**

**D**

**Supplementary figure S2. Alignment of Core amino acid sequences.** Sequences of anti-HBc-/anti-HBs+ OBIs (A), anti-HBc+/anti-HBs+ OBIs (B), anti-HBc+/anti-HBs- OBIs (C), and HBsAg+ non-OBIs (D) were aligned with the consensus sequence derived from HBsAg+ genotype B and genotype C sequences from asymptomatic blood donors. Nucleotides identical to the reference consensus are indicated by dots and deletions by dashes.

**A**

**B**

**C**

**D**

**Supplementary figure S3. Alignment of PreS1/PreS2 amino acid sequences.** Sequences of anti-HBc-/anti-HBs+ OBIs (A), anti-HBc+/anti-HBs+ OBIs (B), anti-HBc+/anti-HBs- OBIs (C), and HBsAg+ non-OBIs (D) were aligned with the consensus sequence derived from HBsAg+ genotype B and genotype C sequences from asymptomatic blood donors. Nucleotides identical to the reference consensus are indicated by dots and deletions by dashes.

**A**

**B**

**C**

**D**

**Supplementary figure S4. Alignment of (partial) S amino acid sequences.** Sequences of anti-HBc-/anti-HBs+ OBIs (A), anti-HBc+/anti-HBs+ OBIs (B), anti-HBc+/anti-HBs- OBIs (C), and HBsAg+ non-OBIs (D) were aligned with the consensus sequence derived from 58 HBsAg+ genotype B and 49 genotype C sequences from asymptomatic blood donors. Nucleotides identical to the reference consensus are indicated by dots and deletions by dashes.

**Supplementary figure S5. Alignment of partial HBx amino acid sequences.** Sequences of 18 anti-HBc-/anti-HBs+ OBIs were aligned with the consensus sequence derived from 28 HBsAg+ genotype C sequences from asymptomatic blood donors. Nucleotides identical to the reference consensus are indicated by dots.
